# Air Pollution Exposure and Muscle Mass and Strength Decline in Older Adults: Results From a Swedish Population–Based Study

**DOI:** 10.1002/jcsm.70111

**Published:** 2025-11-19

**Authors:** Caterina Trevisan, Caterina Gregorio, Anna‐Karin Welmer, Stefano Volpato, Kristina Eneroth, Tom Bellander, Davide Liborio Vetrano, Debora Rizzuto

**Affiliations:** ^1^ Aging Research Center, Department of Neurobiology, Care Sciences and Society Karolinska Institutet and Stockholm University Stockholm Sweden; ^2^ Department of Medical Sciences University of Ferrara Italy; ^3^ Stockholm Gerontology Research Center Stockholm Sweden; ^4^ Division of Physiotherapy, Department of Neurobiology, Care Sciences and Society Karolinska Institutet Stockholm Sweden; ^5^ Women's Health and Allied Health Professionals Theme, Medical Unit Medical Psychology Karolinska University Hospital Stockholm Sweden; ^6^ Environment and Health Administration Stockholm Sweden; ^7^ Institute of Environmental Medicine Karolinska Institutet Stockholm Sweden

**Keywords:** aged, air pollution, muscle strength, probable sarcopenia, prospective study

## Abstract

**Background:**

Emerging evidence suggests that air quality may impact muscle health. However, most studies are limited by cross‐sectional designs or short follow‐ups. We assessed the association of long‐term exposure to ambient air pollutants with changes in muscle mass and strength in older adults.

**Methods:**

We included 3249 participants from the SNAC‐K longitudinal study (mean age 74.3 years; 35.8% males). Muscle strength (measured through handgrip and chair stand tests), muscle mass (derived from calf circumference) and physical performance (assessed through walking speed at a usual pace) were assessed over a 12‐year period. Probable sarcopenia was defined as reduced muscle strength as per the EWGSOP2 criteria. Residential exposure to PM2.5, PM10 and nitrogen oxide (NOx) was estimated for the 5 years preceding baseline. Cox regressions and linear mixed models examined the association of air pollutant exposure with, respectively, probable sarcopenia and longitudinal changes in muscle parameters.

**Results:**

Over 12 years, the cumulative incidence of probable sarcopenia increased with higher exposure (above vs. below the median values) to NOx (36% vs. 28%), PM2.5 (35% vs. 28%) and PM10 (35% vs. 28%). The association between air pollutant levels and the risk of probable sarcopenia was nonlinear (*p*_nonlinearity_ = 0.002 for NOx, 0.001 for PM2.5 and 0.003 for PM10), with an increased risk showing a plateau at very high levels. Higher exposures were associated with an increased risk of developing probable sarcopenia, by 25% for NOx and PM2.5 (HR 95% CI: 1.07–1.47 for both) to 33% (HR 95% CI: 1.14–1.56) for PM10. Elevated pollutant exposure was associated with significantly greater annual declines in lower‐limb strength (chair stand test: 0.40–0.48 s) and walking speed (0.004 m/s).

**Conclusions:**

Long‐term exposure to moderate levels of ambient air pollutants may increase the risk of probable sarcopenia and accelerate declines in lower‐limb strength and physical performance in older adults.

## 1 Introduction

Sarcopenia, characterized by progressive loss of muscle mass and strength, is a major contributor to frailty, disability and loss of independence in older adults, posing a significant public health burden globally [1]. While its development is primarily driven by aging‐related biological changes and lifestyle factors, emerging evidence suggests that environmental exposures may also play a role [2].

The One Health approach emphasizes the interconnection between environmental and human health, highlighting how environmental risk factors can contribute to the development of acute and chronic conditions [3]. Among these, exposure to high levels of air pollution has been linked to adverse health outcomes affecting cardiovascular, respiratory, metabolic and neurological systems [4, 5]. Notably, even very low levels of outdoor air pollutants have been associated with increased risks of nonaccidental mortality and morbidity, suggesting a nonlinear exposure–response relationship [6].

The detrimental effects of air pollution are mediated through mechanisms such as systemic inflammation, oxidative stress and DNA hypomethylation, leading to multisystemic changes including autonomic nervous system dysregulation, endothelial dysfunction and activation of thrombotic pathways [7, 8, 9]. These pathways overlap with those implicated in muscle deterioration, raising concerns about the potential impact of air pollution on muscle health [9, 10].

Recent studies have explored this relationship, showing that exposure to common air pollutants (e.g., PM2.5, PM10, sulphur and nitrogen oxides) is associated with an increased risk of presenting sarcopenia and poorer muscle function [11, 12, 13, 14, 15] [S1–S4]. However, most evidence comes from cross‐sectional studies, while the few longitudinal studies available are limited by short follow‐up periods and often focus on indoor rather than outdoor pollutants [11, 13, 16, 17]. Moreover, little is known about the long‐term effects of air pollution exposure on the decline of both muscle mass and strength in advanced age.

Addressing these gaps, this study investigated the association between long‐term exposure to outdoor air pollutants and the decline in muscle mass and strength in older adults. We hypothesized that individuals with greater exposure to air pollution experience an accelerated deterioration of muscle health in later life.

## 2 Methods

This study is written in accordance with the Strengthening the Reporting of Observational Studies in Epidemiology (STROBE) guidelines (Appendix S1).

### 2.1 Study Population

This study is based on data from the Swedish National Study on Aging and Care in Kungsholmen (SNAC‐K), an ongoing prospective population‐based study involving adults aged ≥ 60 years residing in Kungsholmen, a central area in the city of Stockholm, Sweden. The study population was selected according to random stratified sampling considering the age cohorts of 60, 66, 72, 78, 81, 84, 87, 90, 93, 96 and 99+ years [18]. Based on the participants' age, regular follow‐up assessments were performed every 6 years for the age cohorts 60–72 years and every 3 years for the older age cohorts (age ≥ 78 years) in order to explore the health changes related to aging. The participation rate of the baseline assessment (2001–2004) was 73.3%, and an initial sample of 3363 participants was included in the SNAC‐K. For the current study, 18 individuals were excluded because of missing data on air pollutant exposure, and 96 because of incomplete information on muscle strength at baseline, leading to a final sample of 3249 individuals followed for up to 12 years. Comparison of included and excluded participants (Supplementary Table S1) showed that the latter were more likely to be females, older and physically inactive and to have lower education, occupational level and frequency of risk behaviours. Moreover, they had a higher number of chronic diseases and worse cognitive performance.

From the initial sample of 3249 individuals, we selected the following subsamples based on the purposes of our analyses:

1) subsample of 2034 participants free from probable sarcopenia at baseline, to assess the association between air pollution exposure and the onset of probable sarcopenia during the follow‐up;

2) subsamples of participants with at least two assessments of calf circumference (*n* = 2328), handgrip (*n* = 1848), chair stand test (*n* = 2347) and walking speed (*n* = 2346) from baseline to the 12‐year assessment, to assess the association between air pollutant exposure and changes in these muscle parameters over time.

The SNAC‐K study complies with the principles of the Helsinki Declaration, and the protocol was approved by the regional ethical review board in Stockholm. All participants (or the next of kin, for those with cognitive impairment) gave their written informed consent to take part in the study.

### 2.2 Data Collection

The assessments of the participants at baseline and follow‐ups consisted of personal interviews, administration of validated scales or questionnaires, physical tests and examinations and medical records review.

*Air pollution exposure*. The level of air pollutants at the participants' residential addresses was calculated using Gaussian dispersion modelling with input data from local emission inventories of various source sectors [19]. The mean yearly levels of the following air pollutants were obtained in the 5 years before participants' baseline assessment: nitrogen oxides (NO_x_), that is, the sum of nitrogen monoxide (NO) and nitrogen dioxide (NO_2_), particulate matter with a diameter less than 2.5 μm (PM2.5) or 10 μm (PM10). In Stockholm, the emissions of NO_x_ are dominated by road traffic, with emissions from off‐road machinery, sea traffic and energy facilities as other contributing sources. For PM10, the local contribution mainly consists of road wear particles, but also wood combustion from residential heating. For PM2.5, wood combustion is the largest local emission source, with significant contributions from road wear emissions. Exhaust particles and other combustion particles, although many in number, have very little mass and therefore contribute little to the mass of particles, that is, PM2.5 and PM10. To ensure high resolution in the vicinity of roads, a quadtree receptor grid was used, with 95% of the grid squares within the Kungsholmen district measuring 60 × 60 m^2^ or smaller. For streets bordered by buildings on one or both sides, an additional concentration component was simulated with the Danish Operational Street Pollution model [20]. To obtain total levels of pollutants, annual long‐range contributions, uniform across the model domain, were combined with the model simulations of local PM2.5, PM10 and NOx levels. The long‐range contributions were estimated based on monitoring data at the rural site Norr Malma, located outside the calculation domain, approximately 60 km northeast of Stockholm. The total levels of PM2.5 are primarily influenced by long‐range transport, resulting in a comparatively small relative spatial variability of the modelled PM2.5 level. Comparisons between model‐calculated levels and yearly measurements at three curbside (traffic) monitoring sites and one urban background site in Stockholm City for the period from 1996 to 2004 revealed correlation coefficients of 0.99 for NOx, 0.97 for PM10 and 0.93 for PM2.5.

In this study, the intensity of air pollution exposure was categorized into higher and lower exposure based on the median value: NOx (32 μg/m^3^), PM2.5 (8.29 μg/m^3^) and PM10 (14.7 μg/m^3^).

*Probable sarcopenia and muscle metrics*. Probable sarcopenia, defined as the reduction of muscle strength, was assessed at baseline and follow‐up over 12 years according to the revised criteria published by the European Working Group on Sarcopenia in Older People (EWGSOP2) [1, 21]. In this study, we considered probable sarcopenia as the main outcome, given the consolidated association of reduced muscle strength with adverse outcomes as emerged by the current literature[S5].

Details on the assessment of muscle parameters, including muscle strength, mass and physical performance, are described below.

For muscle strength, we measured the grip strength of both hands separately through the Grippit dynamometer, and the best result was taken for the analyses; in case of missing information on the handgrip, we took into account results from the chair stand test (i.e., recording the time to stand up five times as quickly as possible from a sitting position, without using the hands). According to the recommendations, we used handgrip values < 265 N (corresponding to 27 kg) for men and < 157 N (corresponding to 16 kg) for women or a time for five chair stands > 15 s to detect reduced muscle strength [1].

Muscle mass was estimated through calf circumference. The 20th sex‐specific percentiles of the sample were used as a cutoff to detect low muscle mass, corresponding to < 34 cm for men and < 32 cm for women.

For physical performance, we measured walking speed at usual pace over a 6‐m distance, and a value ≤ 0.8 m/s was considered to detect reduced physical performance. Only for those who defined themselves as slow‐walkers or who performed the test at home, the walking speed test was conducted over 2.4 m.

*Sociodemographic, lifestyle and health‐related information*. For each participant, sociodemographic data including age, sex, education and living arrangement were collected. Concerning lifestyle, we recorded information on smoking habits and alcohol consumption (classified as none or occasional drinker vs. light‐to‐moderate [1–14 drinks/week for men and 1–7 drinks/week for women] vs. heavy [≥15/week for men and ≥8 drinks/week for women]). Moreover, data on the frequency and intensity of physical activities were collected through a structured questionnaire. Participants were defined as physically inactive or active based on their engagement in light or moderate‐to‐intense activities at least once a week over the past 12 months. As a phenotypic criterion of nutritional status, we computed the body mass index (BMI) as the body weight and height squared (kg/m^2^) ratio, measured for each participant.

Cognitive function was evaluated using the Swedish version of the Mini‐Mental State Examination; a score < 28 was used to identify the presence of cognitive impairment, as suggested for highly educated populations [S6]. The history of falls leading to medical attention in the 3 years before baseline was derived from health registers. Moreover, a quantitative measure of multimorbidity was obtained by computing the number of chronic diseases, ascertained by physicians considering the results of physical examinations, biochemical tests, ongoing treatments and medical records included in the national inpatient and outpatient registers [S7]. The complete list of chronic diseases assessed is shown in Appendix S2.

Finally, death dates of participants over the follow‐up were obtained from the Swedish Cause of Death Registry.

### 2.3 Statistical Analysis

Baseline characteristics were summarized using mean and standard deviation (SD), median (interquartile range [IQR]) or frequency (%). Sociodemographic, lifestyle and health‐related data were compared between individuals categorized based on the median air pollutants exposure using ANOVA or the Kruskal–Wallis test for quantitative variables and the chi‐squared or Fisher test for the categorical ones.

Comparison between air pollutant levels in different time periods (5 years before baseline vs. from baseline to the 6‐year follow‐up and from the 6‐ to 12‐year follow‐up) was performed with the paired Student's *t* test.

The incidence rate of probable sarcopenia over the 12‐year follow‐up was computed for individuals with higher versus lower air pollutant exposure and expressed per 1000 person‐years (p‐y). In order to take into account the competing risk of death, we used a cumulative incidence function.

The association of average air pollution levels in the 5 years before baseline, with the risk of developing probable sarcopenia over the 12‐year follow‐up was tested by Cox regression models, adjusted for potential confounders. In particular, Model 1 was adjusted for basic sociodemographic information (age and sex), while Model 2 also included educational level, socioeconomic status and health behaviours (smoking habits and alcohol consumption). In the Cox models, the strength of the associations was expressed as hazard ratios (HRs) and 95% confidence intervals (95% CIs). Missing values in the categorical covariates were included in the model as dummy variables.

A sensitivity analysis was conducted to further account for the competing risk of death, employing a multinomial logistic regression with probable sarcopenia and death (whatever occurred first) as alternative outcomes. The analyses were adjusted for the variables included in Model 2 and expressed the strength of the association as odds ratios (ORs) and 95% CIs.

The association between air pollutant exposure and the changes in muscle parameters (calf circumference, handgrip, chair stand test and walking speed) from baseline to the 12‐year assessment was evaluated through linear mixed models. These analyses included the interaction between air pollution exposure and time (in years) and estimated the average annual change associated with exposure to higher (vs. lower) pollutant levels as β coefficients (with 95% CIs). As a sensitivity analysis, we repeated the linear mixed models after standardizing (by z‐score transformation) the muscle parameters to compare the effect sizes of the association between air pollution exposure and different muscle metrics.

To assess possible effect modification by sociodemographic, lifestyle and health‐related factors in the association between air pollution and probable sarcopenia, we tested the multiplicative interaction for each factor in the Cox regression models and performed appropriate stratified analyses. The following potential modifiers were considered: age (< vs. ≥ 75 years), sex, cognitive function (MMSE < vs. ≥ 28), physical activity level, number of chronic diseases, presence of cardiovascular diseases, respiratory diseases and cancer.

Analyses were performed using R *survival* and *nlme* packages. All tests were two‐tailed, and we set a *p* value < 0.05 for statistical significance.

## 3 Results

The mean age of the total sample was 74.3 (SD 11) years, and 35.8% were males (Table 1). At baseline, over 80% of the sample had at least a high school degree, and around 75% had been white‐collar workers or entrepreneurs. Concerning risk behaviours, around half of the sample was either former (38.3%) or current (14.3%) smokers, and 9.3% reported heavy alcohol consumption. Moreover, 63.6% of those with available data, had a low physical activity level. On average, participants had nearly four chronic diseases and a baseline MMSE score of 27.7. Individuals with higher air pollutant exposures were more likely to be female, older, to have lower education and occupational status, lower alcohol consumption, and to have more chronic diseases than their less‐exposed counterparts. For NOx specifically, those exposed to higher pollutant levels were more likely to be current smokers than those less exposed. Moreover, individuals exposed to higher pollutant levels consistently exhibited worse baseline parameters of muscle mass, strength and physical performance. Consequently, the prevalence of probable sarcopenia was higher in the more exposed group: 40.5% versus 34.3% for NOx, 40.9% versus 33.8% for PM2.5, 41.4% versus 33.4% for PM10. Similar trends were observed when considering the combined presence of low muscle mass and strength.

**TABLE 1:** Characteristics of the sample as a whole and by air pollutants exposure.

		NOx			PM2.5			PM10		
	All	≤32 μg/m^3^	>32 μg/m^3^	*p*	≤8.29 μg/m^3^	>8.29 μg/m^3^	*p*	≤14.7 μg/m^3^	>14.7 μg/m^3^	*p*
*n*	3249	1625	1624		1625	1624		1625	1624	
Sex (male)	1162 (35.8)	627 (38.6)	535 (32.9)	0.001	623 (38.3)	539 (33.2)	0.002	622 (38.3)	540 (33.3)	0.003
Age (years)	74.3 (11.0)	73.2 (10.9)	75.4 (11.0)	<0.001	73.3 (10.8)	75.4 (11.1)	<0.001	73.2 (10.8)	75.5 (11.1)	<0.001
Educational level*				0.002			<0.001			<0.001
Elementary	553 (17.1)	259 (16.0)	294 (18.2)		249 (15.4)	304 (18.9)		241 (14.9)	312 (19.4)	
High school	1596 (49.5)	770 (47.7)	826 (51.3)		774 (47.9)	822 (51.0)		776 (48.0)	820 (50.9)	
University or above	1078 (33.4)	587 (36.3)	491 (30.5)		593 (36.7)	485 (30.1)		599 (37.1)	479 (29.7)	
Occupation*				0.002			<0.001			<0.001
Blue‐collar workers	755 (23.7)	339 (21.2)	416 (26.1)		335 (20.9)	420 (26.4)		339 (21.2)	416 (26.2)	
White‐collar workers	2111 (66.2)	1080 (67.5)	1031 (64.8)		1079 (67.4)	1032 (64.9)		1074 (67.1)	1037 (65.2)	
Entrepreneurs	325 (10.2)	180 (11.3)	145 (9.1)		187 (11.7)	138 (8.7)		188 (11.7)	137 (8.6)	
Smoking habits*				0.006			0.662			0.132
Never	1509 (47.4)	760 (47.7)	749 (47.1)		752 (47.1)	757 (47.7)		754 (47.3)	755 (47.6)	
Former	1219 (38.3)	636 (39.9)	583 (36.7)		622 (39.0)	597 (37.6)		631 (39.6)	588 (37.1)	
Current	454 (14.3)	197 (12.4)	257 (16.2)		221 (13.9)	233 (14.7)		210 (13.2)	244 (15.4)	
Alcohol consumption*				<0.001			<0.001			<0.001
No	1166 (36.6)	532 (33.3)	634 (39.9)		505 (31.6)	661 (41.6)		508 (31.8)	658 (41.4)	
Light‐to‐moderate	1726 (54.1)	902 (56.4)	824 (51.8)		933 (58.3)	793 (49.9)		934 (58.4)	792 (49.9)	
Heavy	296 (9.3)	164 (10.3)	132 (8.3)		161 (10.1)	135 (8.5)		158 (9.9)	138 (8.7)	
High physical activity level*	658 (24.2)	372 (26.9)	286 (21.4)	0.001	366 (26.1)	292 (22.1)	0.015	372 (26.5)	286 (21.7)	0.004
No. chronic diseases	3.99 (2.46)	3.81 (2.43)	4.16 (2.48)	<0.001	3.79 (2.42)	4.18 (2.49)	<0.001	3.76 (2.38)	4.22 (2.52)	<0.001
MMSE*	27.71 (4.48)	27.83 (4.46)	27.60 (4.50)	0.145	27.93 (4.28)	27.49 (4.67)	0.005	27.95 (4.24)	27.47 (4.70)	0.002
Fall history	260 (8.0)	107 (6.6)	153 (9.4)	0.004	106 (6.5)	154 (9.5)	0.002	108 (6.6)	152 (9.4)	0.005
Calf circumference (cm)*	35.8 (3.8)	36.1 (3.7)	35.6 (3.8)	<0.001	36.0 (3.7)	35.6 (3.9)	0.001	36.1 (3.6)	35.6 (3.9)	<0.001
Handgrip (N)*	255.8 (113.8)	264.4 (117.8)	246.5 (108.6)	<0.001	263.8 (118.2)	247.0 (108.0)	<0.001	264.3 (117.7)	246.5 (108.6)	<0.001
Chair stand test (sec)*	28.84 (27.18)	26.93 (26.50)	30.75 (27.73)	<0.001	26.71 (26.20)	30.97 (27.98)	<0.001	26.56 (26.05)	31.13 (28.09)	<0.001
Walking speed (m/s)*	0.96 (0.47)	1.00 (0.47)	0.92 (0.47)	<0.001	1.00 (0.46)	0.92 (0.48)	<0.001	1.01 (0.46)	0.91 (0.48)	<0.001
Probable sarcopenia	1215 (37.4)	558 (34.3)	657 (40.5)	<0.001	550 (33.8)	665 (40.9)	<0.001	543 (33.4)	672 (41.4)	<0.001
Combined low muscle strength and mass*	309 (9.6)	133 (8.3)	176 (11.0)	0.011	135 (8.4)	174 (10.9)	0.023	131 (8.2)	178 (11.1)	0.006

*Note:* *p* values refer to the comparison between individuals with different air pollutant exposure.

Abbreviations: MMSE, Mini‐Mental State Examination; NOx, nitrogen oxides; PM2.5, particular matter with a diameter not exceeding 2.5 μm; PM10, particular matter with a diameter not exceeding 10 μm.

* *n* = 22 participants had missing data on education, *n* = 58 on occupation, *n* = 67 on smoking habit, *n* = 61 on drinking habit, *n* = 526 on physical activity level, *n* = 24 on MMSE, *n* = 55 on calf circumference, *n* = 648 on handgrip, *n* = 7 on chair stand test, *n* = 28 on walking speed and *n* = 46 on combined low muscle strength and mass.

A gradual reduction in air pollutant levels was observed from the prebaseline period over the 12‐year follow‐up. This decreasing trend was found both in participants with and without probable sarcopenia (Supplementary Table S2). Over the 12‐year follow‐up, among the 2034 participants with normal muscle strength at baseline, 637 (31.3%) developed probable sarcopenia. The incidence rate of probable sarcopenia increased from those less exposed to those more exposed to NOx (32.1 vs. 43 per 1000 p‐y), PM2.5 (32.4 vs. 42.8 per 1000 p‐y) and PM10 (32.1 vs. 43.3 per 1000 p‐y) (*p* < 0.001 for all comparisons). This picture was confirmed by the cumulative incidence function (Supplementary Figure S1).

**FIGURE 1:**
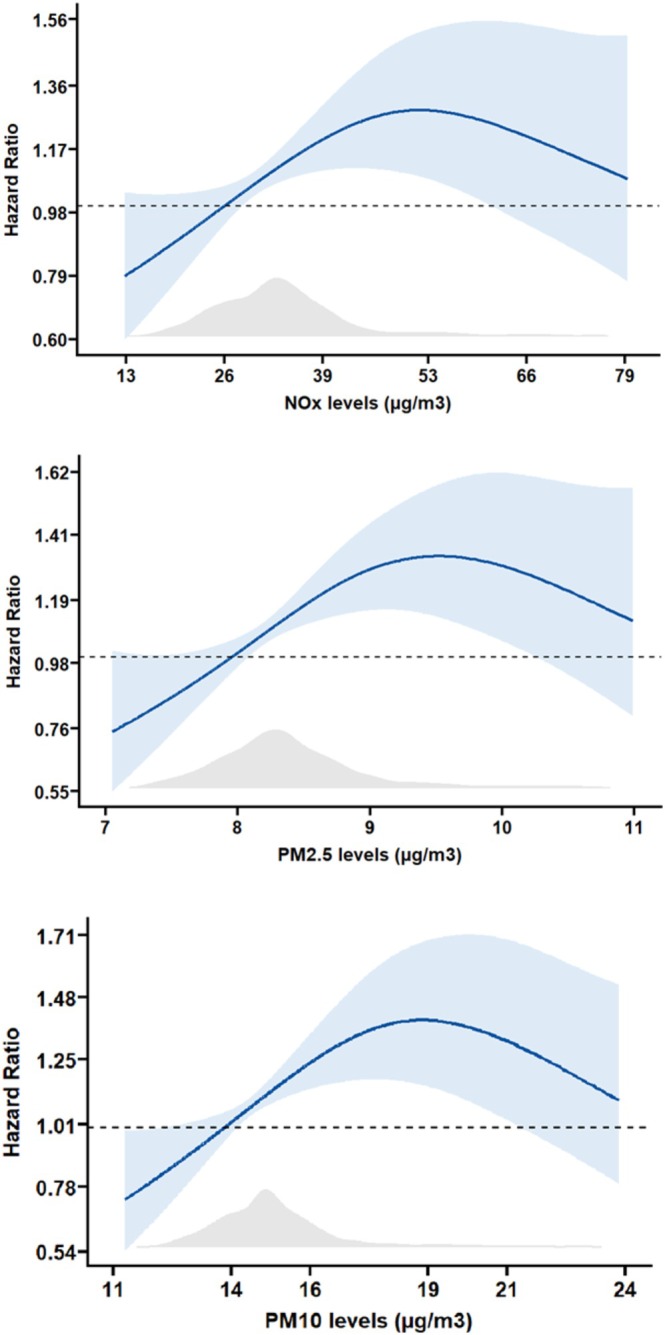
Dose–response association between air pollutant levels and the risk of probable sarcopenia. *Notes*. Hazard ratios refer to the risk of probable sarcopenia over the 12‐year follow‐up. The blue area indicates the 95% confidence interval of hazard ratios, and the grey area indicates the frequency of air pollutant levels in the study sample. *p* values for the association between air pollutants and the risk of probable sarcopenia are: for NOx, *p* value = 0.20 (overall), *p* value = 0.002 (nonlinearity); for PM2.5, *p* value = 0.06 (overall), *p* value = 0.001 (nonlinearity); for PM10, *p* value = 0.18 (overall), *p* value = 0.003 (nonlinearity).

The association between NOx, PM2.5 and PM10 levels and the risk of probable sarcopenia was nonlinear (Figure 1). Cox regression analysis (Table 2), adjusted for potential confounders, showed that exposure levels above the median level of NOx, PM2.5 and PM10 were associated with a risk of developing probable sarcopenia ranging from 25% for NOx and PM2.5 (95% CI: 1.07–1.47 for both) to 33% (95% CI: 1.14–1.56) for PM10. Similar results were observed at multinomial logistic regression (Supplementary Table S3).

**TABLE 2:** Cox regression for the association between air pollutant exposure and probable sarcopenia over the 12‐year follow‐up (*N* = 2034).

Mean annual air pollution exposure	HR (95% CI) of probable sarcopenia, *p*	
	Model 1	Model 2
**NOx**		
≤32 μg/m^3^	[ref]	[ref]
>32 μg/m^3^	1.27 (1.09, 1.49), *p* = 0.002	1.25 (1.07, 1.47), *p* = 0.005
**PM2.5**		
≤8.29 μg/m^3^	[ref]	[ref]
>8.29 μg/m^3^	1.26 (1.08, 1.48), *p* = 0.003	1.25 (1.07, 1.47), *p* = 0.005
**PM10**		
≤14.7 μg/m^3^	[ref]	[ref]
>14.7 μg/m^3^	1.35 (1.15, 1.57), *p* < 0.001	1.33 (1.14, 1.56). *p* < 0.001

*Note:* Model 1 is adjusted for age and sex; Model 2 is also adjusted for educational level, occupation, smoking habits and alcohol consumption.

Abbreviations: 95% CI, 95% confidence interval; HR, hazard ratio; NOx, nitrogen oxides; PM2.5, particulate matter with a diameter not exceeding 2.5 μm; PM10, particulate matter with a diameter not exceeding 10 μm.

The association between higher air pollutant exposure and the risk of probable sarcopenia was more marked among the subgroups of participants with a high physical activity level, lower cognitive performance, and those with respiratory diseases (*p* for interaction < 0.10). Despite the absence of significant statistical interactions, for all pollutants, associations with probable sarcopenia were more marked in the older ones (Supplementary Table S4).

When evaluating the mean changes of muscle parameters by air pollution at linear mixed models, we found that individuals more exposed to PM10 had a steeper reduction in calf circumference than those less exposed (−0.03 cm/year, 95% CI: −0.05, −0.004) (Table 3). Higher exposure to all air pollutants was associated with a greater worsening in the chair stand test, with a mean increase ranging from 0.40 to 0.48 s per year in the time needed to perform the test. Moreover, for the three high‐exposure groups, the annual reduction in walking speed exceeded by 0.004 m/s that of the less exposed participants. After z‐score transformation of the outcomes, greater effect sizes for the associations between air pollutants and muscle parameter changes were observed for the chair stand and walking speed tests (Supplementary Table S5).

**TABLE 3:** Difference according to exposure level in changes in calf circumference, handgrip, chair stand test and walking speed test results over 12 years (linear mixed models).

	Difference in change by year (95% CI)*, p*		
	NOx (> vs. ≤ 32 μg/m^3^)	PM2.5 (> vs. ≤ 8.29 μg/m^3^)	PM10 (> vs. ≤ 14.7 μg/m^3^)
Calf circumference (cm/year; *n* = 2328)			
*Model 1*	−0.01 (−0.03, 0.01) *p* = 0.207	−0.02 (−0.04, 0.001) *p* = 0.058	−0.03 (−0.05, −0.004) *p* = 0.017
*Model 2*	−0.01 (−0.03, 0.01) *p* = 0.217	−0.02 (−0.04, 0.001) *p* = 0.059	−0.03 (−0.05, −0.004) *p* = 0.017
Handgrip (N/year; *n* = 1848)			
*Model 1*	−0.30 (−0.75, 0.14) *p* = 0.179	−0.06 (−0.50, 0.38) *p* = 0.791	−0.33 (−0.77, 0.12) *p* = 0.150
*Model 2*	−0.30 (−0.75, 0.14) *p* = 0.179	−0.06 (−0.50, 0.39) *p* = 0.795	−0.33 (−0.77, 0.12) *p* = 0.150
Chair stand test (s/year; *n* = 2347)			
*Model 1*	0.40 (0.18, 0.62) *p* < 0.001	0.39 (0.16, 0.61) *p* = 0.001	0.47 (0.24, 0.69) *p* < 0.001
*Model 2*	0.41 (0.19, 0.64) *p* < 0.001	0.40 (0.17, 0.62) *p* < 0.001	0.48 (0.26, 0.70) *p* < 0.001
Walking speed (m/s/year; *n* = 2346)			
*Model 1*	−0.004 (−0.01, −0.001) *p* = 0.008	−0.004 (−0.01, −0.001) *p* = 0.008	−0.004 (−0.01, −0.001) *p* = 0.012
*Model 2*	−0.004 (−0.01, −0.001) *p* = 0.006	−0.004 (−0.01, −0.001) *p* = 0.006	−0.004 (−0.01, −0.001) *p* = 0.008

*Note:* Model 1 is adjusted for age and sex; Model 2 is adjusted also for educational level, occupation, smoking habits and alcohol consumption. Time is included in the model in years, so that the β coefficients represent the difference between the mean annual change in muscle parameters of participants with higher versus lower pollutant exposures.

Abbreviations: 95% CI, 95% confidence interval; NOx, nitrogen oxides; PM2.5, particulate matter with a diameter not exceeding 2.5 μm; PM10, particulate matter with a diameter not exceeding 10 μm..

## 4 Discussion

This study found that moderate air pollutant exposure was associated with a higher risk of probable sarcopenia and accelerated deterioration, especially in lower limb muscle strength and physical performance. These findings align with the existing literature, which highlights the detrimental effect of indoor and outdoor air pollutants on outcomes related to muscle health [11, 12, 13, 16] [S8, S9] and, most recently, functional disability [22, 23].

Interestingly, the relationship between air pollution level and the risk of probable sarcopenia in our cohort showed an apparent plateau at very high pollutant concentrations. This trend has been previously observed in studies focusing on the association between air pollution and other health‐related outcomes [24, 25], and may be explained by several reasons. Among these, the small number of participants exposed to higher levels of air pollutants and possible survival bias may play a role in reducing the statistical power of the analysis and buffering the expected dose–effect relationship, respectively. Moreover, we tested a binary outcome rather than a continuous one, so that our curves might suggest that even low air pollutant concentrations are sufficient to trigger muscle damage, leading to a progressive loss of muscle strength.

The detrimental impact of air pollutants on muscle health is primarily mediated by inflammation and oxidative stress, two interconnected mechanisms. Exposure to pollutants such as NOx and particulate matter triggers an inflammatory response via deposition in the respiratory tract, activation of alveolar macrophages and systemic release of cytokines like interleukin‐6 and tumour necrosis factor‐α [9, 26, 27]. Chronic low‐grade inflammation, in turn, promotes muscle catabolism and inhibits anabolic processes, contributing to sarcopenia development [10]. In parallel, air pollution increases reactive oxygen species (ROS) production while impairing antioxidant defences, leading to oxidative stress, mitochondrial dysfunction and apoptosis in muscle cells that, overall, affect muscle mass, power and injury repair [10, 28]. Moreover, ROS accumulation may also affect the neuromuscular junction, which plays a central role in muscle contraction efficiency [29]. In addition to the direct damage to muscle fibres, these processes further exacerbate inflammation, creating a vicious cycle [27, 28].

Finally, an additional aspect to consider when studying the association between air pollution and sarcopenia is physical activity level. Indeed, greater engagement in physical activity may be linked to higher exposure to outdoor air pollutants. This hypothesis is supported by our findings because the relationship between air pollution and probable sarcopenia was stronger among the most active individuals. However, physical activity may also be a mediator in the studied association. In fact, the adverse cardiovascular, respiratory and metabolic effects linked to air pollution exposure may lead to physical inactivity, which negatively affects muscle health and individual physical performance [30].

The pro‐inflammatory and pro‐oxidative effects of air pollution exposure could be exacerbated in the presence of conditions already characterized by persistent inflammatory and oxidative states. Accordingly, we found that the detrimental effect of air pollution exposure on muscle strength was more marked among people with cancer and respiratory diseases, which have been linked with a chronic inflammatory status and a higher risk of sarcopenia [31]. Therefore, these subgroups of individuals may be more vulnerable to further inflammatory and pro‐oxidative effects of air pollutants.

When looking at single muscle metrics, we found that individuals with higher exposure to any pollutant were more likely to present a steeper decline in muscle strength in the lower limbs and walking speed. This finding is of great interest since these tests, especially walking, require not only musculoskeletal efficiency but also neurological competencies (for coordinating motor activities) and cardiopulmonary fitness. Therefore, the significant effect of air pollution exposure on these outcomes can be attributed to the cumulative detrimental effects on the neurological [32] and cardiometabolic systems [4] and on skeletal muscle health. These findings also align with our subgroup analyses, as greater effect sizes for the association between air pollution exposure and probable sarcopenia emerged among participants with cognitive deficits, who could also present dysfunctions in coordination and movement mechanics affecting muscle efficiency [33]. Of note, the differential extent of walking speed decline in participants exposed to higher air pollutant levels was 0.004 m/s, smaller than the most common minimally meaningful changes, ranging between 0.03 and 0.1 m/s [34, 35]. However, this value adds to the rate of decline in walking speed related to several factors, such as increasing age, unhealthy behaviours and acute and chronic comorbidities. Therefore, from a broader perspective, the impact of this modifiable factor on a population level may be significant, as it may contribute to the acceleration of physical performance decline and loss of self‐sufficiency among older adults, with relevant implications for healthcare and social systems.

Finally, it is worth noting that, among the pollutants considered in our study, PM10 was more strongly associated with probable sarcopenia than PM2.5 and NOx. Moreover, individuals with higher exposure to PM10 presented a steeper decline in muscle mass. A stronger effect of PM10 on muscle parameters was also observed in previous European studies [14], but not in other works mostly performed in Chinese areas [12, 15]. Although this result needs to be further explored, PM10 has been suggested to be more associated with the atherosclerotic process and the development of peripheral artery diseases than other pollutants [36]. Therefore, it may further promote the loss of muscle mass and function through vascular damage.

Among the limitations of the study, we recognize that calf circumference is only a proxy for muscle mass and its measurement is not the most precise method to detect low muscle mass, although it can be used in contexts where no other parameters are available [1, 37]. In our study, considering calf circumference may have led to underestimating the number of cases with combined low muscle mass and strength, as well as the relationship between air pollution exposure and muscle mass changes. Instead, this issue did not affect our main findings, which focused on the development of probable sarcopenia, namely, the onset of low muscle strength, irrespective of the presence of low muscle mass. As concerns handgrip strength, we recognize that our results on muscle metrics changes could have been affected by the smaller sample size in that analysis due to missing values, which have led to lower statistical power. When interpreting our data, we should also consider the possible influence of survivorship bias. Indeed, individuals more exposed to air pollutants had a higher prevalence of probable sarcopenia at baseline; therefore, the evaluation of the association between air pollution and incident probable sarcopenia over the follow‐up could have been underestimated. Moreover, our study focuses on the Stockholm area, which may not be representative of other geographical contexts in terms of both air pollutant exposure and population characteristics. Concerning air pollution, Sweden has implemented environmental policies aimed at improving air quality in recent decades, making it a low‐emission country [38]. Nonetheless, the median annual values of air pollutants in the considered time interval exceed the recommended WHO targets, especially for PM2.5 (5 μg/m^3^) and NO2 (10 μg/m^3^)^3^ [39], and recent data show that a not negligible part of the population is still exposed to levels above those thresholds [38]. We acknowledge that potential changes in the residential location of the study participants during the observation period have not been considered in our analyses. However, previous studies suggest that residential moves are relatively uncommon among older individuals, with a frequency substantially decreasing after the age of 66–67 [40]. Regarding the cohort characteristics, the SNAC‐K participants, on average, have high educational and socioeconomic levels, which may influence exposure to both air pollution and the risk factors of sarcopenia. Overall, the above aspects may affect the generalizability of our findings to other populations but are more likely to underestimate than overestimate the tested associations. Finally, despite adjusting our models for sociodemographic characteristics and some unhealthy behaviours, we did not consider other potential confounders. However, only weak attenuation of the estimates was observed in the multivariable models, so we do not expect residual confounding to substantially affect our findings. On the other hand, the large cohort and the prospective study design are strengths of our study. Moreover, the assessment of muscle strength and physical performance through objective and validated measures over a long follow‐up further reinforces the study findings. Notably, the study's observational design does not allow us to draw any causal inference about the relationship between air pollution and probable sarcopenia. Moreover, it did not assess the pathophysiological mechanisms that may link air pollution exposure to the worsening of muscle parameters; this issue will be the focus of future investigations.

In conclusion, our findings suggest that even moderate levels of urban air pollution are associated with accelerated decline in muscle health, especially muscle strength, among older adults. This supports the importance of strict air quality regulations to maintain functional independence in aging populations. Further studies are warranted to confirm causality and to elucidate underlying biological mechanisms.

## Author Contributions

Caterina Trevisan, Tom Bellander, Stefano Volpato, Davide Liborio Vetrano and Debora Rizzuto contributed to the conception of the work. Caterina Trevisan, Caterina Gregorio, Anna‐Karin Welmer, Kristina Eneroth, Tom Bellander, Davide Liborio Vetrano and Debora Rizzuto contributed to the acquisition of the data. Caterina Trevisan, Caterina Gregorio, Kristina Eneroth and Debora Rizzuto contributed to the data analysis. All authors contributed to the interpretation of data for the work. Caterina Trevisan drafted the work. Caterina Gregorio, Anna‐Karin Welmer, Stefano Volpato, Kristina Eneroth, Tom Bellander, Davide Liborio Vetrano and Debora Rizzuto reviewed the manuscript critically for important intellectual content. All authors gave final approval for the version to be published and agreed to be accountable for all aspects of the work.

## Conflicts of Interest

The authors declare no conflicts of interest.

## Supporting information


**Appendix S1:** STROBE statement—checklist of items that should be included in reports of observational studies.
**Table S1:** Characteristics of participants included and excluded from the analytical sample.
**Figure S1:** Cumulative incidence function curves for the association of NOx, PM2.5 and PM10 with probable sarcopenia over the 12‐year follow‐up.
**Table S2:** Average air pollutant levels from the pre‐baseline period to the 12‐year follow‐up.
**Table S3:** Multinomial logistic regression for the association of air pollutant exposure with probable sarcopenia and death (alternative outcomes) over 12 years.
**Table S4:** Subgroup Cox regression analyses for the association between air pollutant exposure and probable sarcopenia over the 12‐year follow‐up.
**Table S5:** Difference according to exposure level in changes in standardized calf circumference, handgrip, chair stand test and walking speed test results over 12 years (linear mixed models).
