# Supplementary material for: Air Pollution Exposure and Muscle Mass and Strength Decline in Older Adults: Results From a Swedish Population–Based Study
Source: J Cachexia Sarcopenia Muscle. 2025 Nov 19;16(6):e70111. doi: 10.1002/jcsm.70111 (PMC12629866; doi:10.1002/jcsm.70111)
Supplement: Supplementary file 1 — Appendix S1: STROBE statement—checklist of items that should be included in reports of observational studies. Table S1: Characteristics of participants included and excluded from the analytical sample. Figure S1: Cumulative incidence function curves for the association of NOx, PM2.5 and PM10 with probable sarcopenia over the 12‐year follow‐up. Table S2: Average air pollutant levels from the pre‐baseline period to the 12‐year follow‐up. Table S3: Multinomial logistic regression for the association of air pollutant exposure with probable sarcopenia and death (alternative outcomes) over 12 years. Table S4: Subgroup Cox regression analyses for the association between air pollutant exposure and probable sarcopenia over the 12‐year follow‐up. Table S5: Difference according to exposure level in changes in standardized calf circumference, handgrip, chair stand test and walking speed test results over 12 years (linear mixed models). [file JCSM-16-e70111-s001.doc]

**SUPPLEMENTARY MATERIAL**

**Appendix 1. STROBE Statement—checklist of items that should be included in r**eports of observational studies

|  | Item No | Recommendation | page |
| --- | --- | --- | --- |
| **Title and abstract** | 1 | (*a*) Indicate the study’s design with a commonly used term in the title or the abstract | 2 |
|  |  | (*b*) Provide in the abstract an informative and balanced summary of what was done and what was found | 2 |
| Introduction | | |  |
| Background/rationale | 2 | Explain the scientific background and rationale for the investigation being reported | 3 |
| Objectives | 3 | State specific objectives, including any prespecified hypotheses | 3 |
| Methods | | |  |
| Study design | 4 | Present key elements of study design early in the paper | 4 |
| Setting | 5 | Describe the setting, locations, and relevant dates, including periods of recruitment, exposure, follow-up, and data collection | 4 |
| Participants | 6 | (*a*) *Cohort study*—Give the eligibility criteria, and the sources and methods of selection of participants. Describe methods of follow-up | 4 |
|  |  | (*b*) *Cohort study*—For matched studies, give matching criteria and number of exposed and unexposed | NA |
| Variables | 7 | Clearly define all outcomes, exposures, predictors, potential confounders, and effect modifiers. Give diagnostic criteria, if applicable | 4-7 |
| Data sources/ measurement | 8 | For each variable of interest, give sources of data and details of methods of assessment (measurement). Describe comparability of assessment methods if there is more than one group | 4-7 |
| Bias | 9 | Describe any efforts to address potential sources of bias | 7-8 |
| Study size | 10 | Explain how the study size was arrived at | 4 |
| Quantitative variables | 11 | Explain how quantitative variables were handled in the analyses. If applicable, describe which groupings were chosen and why | 4-7 |
| Statistical methods | 12 | (*a*) Describe all statistical methods, including those used to control for confounding | 7-8 |
|  |  | (*b*) Describe any methods used to examine subgroups and interactions | 8 |
|  |  | (*c*) Explain how missing data were addressed | 4, 8 |
|  |  | (*d*) *Cohort study*—If applicable, explain how loss to follow-up was addressed | 4 |
|  |  | (*e*) Describe any sensitivity analyses | 8 |

| Results | | |  |
| --- | --- | --- | --- |
| Participants | 13* | (a) Report numbers of individuals at each stage of study—eg numbers potentially eligible, examined for eligibility, confirmed eligible, included in the study, completing follow-up, and analysed | 4 |
|  |  | (b) Give reasons for non-participation at each stage | 4 |
|  |  | (c) Consider use of a flow diagram | NA |
| Descriptive data | 14 | (a) Give characteristics of study participants (eg demographic, clinical, social) and information on exposures and potential confounders | 8-9 |
|  |  | (b) Indicate number of participants with missing data for each variable of interest | 4 |
|  |  | (c) *Cohort study*—Summarise follow-up time (eg, average and total amount) | 8-9 |
| Outcome data | 15 | *Cohort study*—Report numbers of outcome events or summary measures over time | 9-10 |
|  |  | *Case-control study—*Report numbers in each exposure category, or summary measures of exposure | NA |
|  |  | *Cross-sectional study—*Report numbers of outcome events or summary measures | NA |
| Main results | 16 | (*a*) Give unadjusted estimates and, if applicable, confounder-adjusted estimates and their precision (eg, 95% confidence interval). Make clear which confounders were adjusted for and why they were included | 9 |
|  |  | (*b*) Report category boundaries when continuous variables were categorized | 9 |
|  |  | (*c*) If relevant, consider translating estimates of relative risk into absolute risk for a meaningful time period | 9 |
| Other analyses | 17 | Report other analyses done—eg analyses of subgroups and interactions, and sensitivity analyses | 9-10 |
| Discussion | | |  |
| Key results | 18 | Summarise key results with reference to study objectives | 10 |
| Limitations | 19 | Discuss limitations of the study, taking into account sources of potential bias or imprecision. Discuss both direction and magnitude of any potential bias | 12 |
| Interpretation | 20 | Give a cautious overall interpretation of results considering objectives, limitations, multiplicity of analyses, results from similar studies, and other relevant evidence | 10-12 |
| Generalisability | 21 | Discuss the generalisability (external validity) of the study results | 10-12 |
| Other information | | |  |
| Funding | 22 | Give the source of funding and the role of the funders for the present study and, if applicable, for the original study on which the present article is based | 2, 13 |

Supplementary Table 1. Characteristics of participants included and excluded from the analytical sample

|  | **Included** | **Excluded** | **p-value** |
| --- | --- | --- | --- |
| n | 3249 | 114 |  |
| Sex (male) | 1162 (35.8) | 19 (16.7) | <0.001 |
| Age (years) | 74.31 (10.99) | 87.52 (9.68) | <0.001 |
| Educational level* |  |  | <0.001 |
| Elementary | 553 (17.0) | 36 (31.6) |  |
| High school | 1596 (49.1) | 55 (48.2) |  |
| University or above | 1078 (33.2) | 12 (10.5) |  |
| Missing | 22 (0.7) | 11 (9.6) |  |
| Occupation* |  |  | <0.001 |
| Blue-collar workers | 755 (23.2) | 37 (32.5) |  |
| White-collar workers | 2111 (65.0) | 44 (38.6) |  |
| Entrepreneurs | 325 (10.0) | 7 (6.1) |  |
| Missing | 58 (1.8) | 26 (22.8) |  |
| Smoking habits* |  |  | <0.001 |
| Never | 1509 (46.4) | 43 (37.7) |  |
| Former | 1219 (37.5) | 24 (21.1) |  |
| Current | 454 (14.0) | 11 (9.6) |  |
| Missing | 67 (2.1) | 36 (31.6) |  |
| Alcohol consumption* |  |  | <0.001 |
| No | 1166 (35.9) | 61 (53.5) |  |
| Light-to-moderate | 1726 (53.1) | 18 (15.8) |  |
| Heavy | 296 (9.1) | 4 (3.5) |  |
| Missing | 61 (1.9) | 31 (27.2) |  |
| Physical activity level* |  |  | <0.001 |
| No or mild | 2065 (63.6) | 23 (20.2) |  |
| High | 658 (20.3) | 2 (1.8) |  |
| Missing | 526 (16.2) | 89 (78.1) |  |
| N. chronic diseases | 3.99 (2.46) | 5.51 (2.68) | <0.001 |
| Mini-Mental State Examination* | 27.67 (4.50) | 14.07 (10.96) | <0.001 |
| Fall history | 260 (8.0) | 32 (28.1) | <0.001 |

*Notes*. P-values refer to the comparison between individuals with different air pollutant exposure. *n=22 participants had missing data on education, n=58 on occupation, n=67 on smoking habit, n=61 on drinking habit, n=526 on physical activity level, n=24 on Mini-Mental State Examination, n=55 on calf circumference, n=648 on handgrip, n=7 on chair stand test, n=28 on walking speed, and n=46 on confirmed sarcopenia.

**Appendix 2. List of chronic diseases considered**

The following chronic diseases were assessed by physicians from physical examination, blood tests, review of medicines used, and national health registers[1]: anemia; venous and lymphatic diseases; blood and blood forming organ diseases; autoimmune diseases; thyroid diseases; hypertension; ischemic heart disease; heart failure; peripheral vascular disease; atrial fibrillation; cardiac valve diseases; bradycardias and conduction diseases, other cardiovascular diseases; chronic infectious diseases, chronic kidney diseases; other genitourinary diseases; chronic pancreas, biliary tract and gallbladder diseases; colitis and related diseases; allergy; asthma; COPD, emphysema, chronic bronchitis; other respiratory diseases; diabetes; esophagus, stomach and duodenum diseases; colitis and related diseases; inflammatory bowel diseases; other digestive diseases; hypercholesterolemia; obesity; chronic liver diseases; other metabolic diseases; blindness, visual impairment; cataract and other lens diseases; glaucoma; other eye diseases; deafness, hearing impairment; ear, nose, throat diseases; chronic ulcer of the skin; other skin diseases; inflammatory arthropathies; osteoarthritis and other degenerative joint diseases; osteoporosis; dorsopathies; migraine and facial pain syndromes; neurotic, stress-related and somatoform diseases; cerebrovascular disease; other neurological diseases; multiple sclerosis; peripheral neuropathy; epilepsy; sleep disorders; depression and mood diseases; other psychiatric and behavioral diseases; schizophrenia and delusional diseases; Parkinson and parkinsonism; prostate diseases; solid neoplasms; hematological neoplasms; and, chromosomal abnormalities.

**Supplementary Table 2. Average air pollutant levels from the pre-baseline period to the 12-year follow-up**

|  | **All** | **No sarcopenia** | **Probable sarcopenia** | **P-value*** |
| --- | --- | --- | --- | --- |
| **NOx** |  |  |  |  |
| 5 years before baseline | 33.62 (11.78) | 33.38 (12.48) | 34.02 (10.49) | 0.133 |
| Baseline to 6-year follow-up | 25.77 (8.18) | 25.49 (8.72) | 26.19 (7.18) | 0.062 |
| 6- to 12-year follow-up | 19.37 (6.34) | 19.06 (6.53) | 19.84 (6.02) | 0.032 |
| **PM2.5** |  |  |  |  |
| 5 years before baseline | 8.39 (0.70) | 8.37 (0.73) | 8.42 (0.63) | 0.029 |
| Baseline to 6-year follow-up | 8.52 (0.60) | 8.50 (0.63) | 8.56 (0.56) | 0.027 |
| 6- to 12-year follow-up | 6.90 (0.47) | 6.89 (0.49) | 6.93 (0.45) | 0.148 |
| **PM10** |  |  |  |  |
| 5 years before baseline | 15.12 (2.18) | 15.08 (2.31) | 15.19 (1.95) | 0.161 |
| Baseline to 6-year follow-up | 15.22 (1.92) | 15.16 (2.04) | 15.32 (1.70) | 0.068 |
| 6- to 12-year follow-up | 14.32 (1.66) | 14.26 (1.68) | 14.41 (1.62) | 0.118 |

*Notes*. Values are mean (standard deviation). Air pollutant levels compared between the three time periods were statistically significant (p<0.001) at the paired Student t-test. *P-values refer to the comparison between participants with vs without probable sarcopenia at baseline (for 5 years before baseline), the 6-year follow-up (for the period from baseline to 6-year follow-up), and the 12-year follow-up (for the period from 6- to 12-year follow-up).

**Supplementary Figure 1. Cumulative incidence function curves for the association of NOx, PM2.5, and PM10 with probable sarcopenia over the 12-year follow-up**


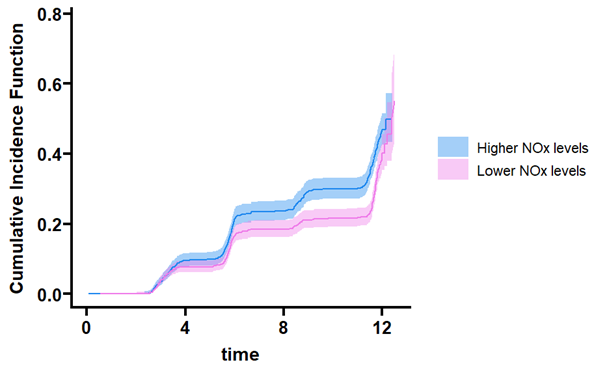


*
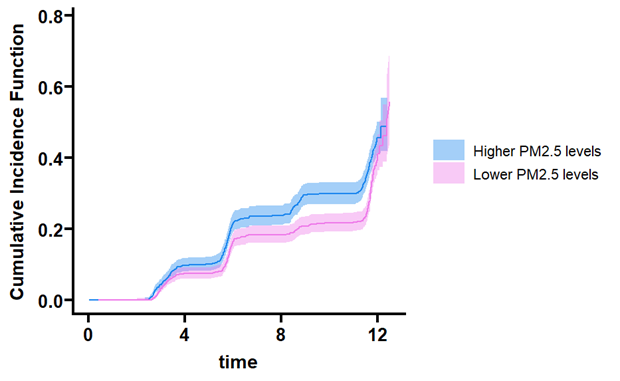
*

*
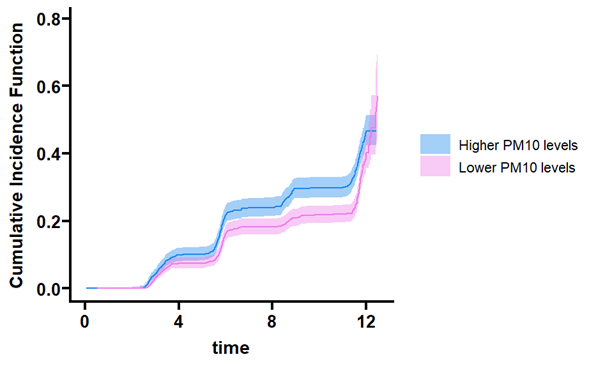
*

*Abbreviations*: PM2.5, Particular Matter with a diameter not exceeding 2.5 μm; PM10, Particular Matter with a diameter not exceeding 10 μm. *Notes*. High and low levels of air pollutants were defined based on median values: 32 μg/m^3^ (NOx); 8.29 μg/m^3^ (PM2.5); 14.7 μg/m^3^ (PM10).

**Supplementary Table 3. Multinomial logistic regression for the association of air pollutant exposure with probable sarcopenia and death (alternative outcomes) over 12 years**

| **Mean annual air pollution exposure** | **Odds Ratio (95% Confidence Interval), *p-value*** | |
| --- | --- | --- |
|  | **Probable sarcopenia** | **Death** |
| **NOx** |  |  |
| ≤32 μg/m^3^ | [ref] | [ref] |
| >32 μg/m^3^ | 1.26 (1.01, 1.57), p=0.042 | 0.85 (0.64, 1.12), p=0.300 |
| **PM2.5** |  |  |
| ≤8.29 μg/m^3^ | [ref] | [ref] |
| >8.29 μg/m^3^ | 1.31 (1.05, 1.64), p=0.017 | 0.99 (0.75, 1.31), p=0.900 |
| **PM10** |  |  |
| ≤14.7 μg/m^3^ | [ref] | [ref] |
| >14.7 μg/m^3^ | 1.39 (1.12, 1.74), p=0.004 | 1.11 (0.84, 1.47), p=0.400 |

*Abbreviations*: NOx, nitrogen oxides; PM2.5, Particular Matter with a diameter not exceeding 2.5 μm; PM10, Particular Matter with a diameter not exceeding 10 μm. *Notes*. Model is adjusted for age, sex, educational level, occupation, smoking habits, and alcohol consumption.

**Supplementary Table 4. Subgroup Cox regression analyses for the association between air pollutant exposure and probable sarcopenia** over the 12-year follow-up

|  |  | **HR (95%CI) of probable sarcopenia**  ***p-value*** | | |
| --- | --- | --- | --- | --- |
|  |  | **NOx**  **(> vs ≤32 μg/m^3^)** | **PM2.5**  **(> vs ≤8.29 μg/m^3^)** | **PM10**  **(> vs ≤14.7 μg/m^3^)** |
| Sex |  | p*_interaction_*= 0.442 | p*_interaction_*= 0.875 | p*_interaction_*= 0.403 |
| Females | 1169 | 1.21 (0.99, 1.47) | 1.27 (1.04, 1.54) | 1.41 (1.15, 1.71) |
| Males | 865 | 1.38 (1.06, 1.80) | 1.20 (0.92, 1.58) | 1.17 (0.89, 1.53) |
| Age |  | p*_interaction_*= 0.411 | p*_interaction_*= 0.378 | p*_interaction_*= 0.302 |
| <75 | 1526 | 1.16 (0.94, 1.45) | 1.19 (0.96, 1.49) | 1.25 (1.01, 1.56) |
| ≥75 | 508 | 1.25 (0.99, 1.58) | 1.37 (1.09, 1.74) | 1.45 (1.15, 1.84) |
| Occupation |  | p*_interaction_*= 0.325 | p*_interaction_*= 0.616 | p*_interaction_*= 0.434 |
| Blue-collar workers | 382 | 1.10 (0.79, 1.54) | 1.18 (0.84, 1.65) | 1.44 (1.02, 2.03) |
| White-collar workers | 1414 | 1.26 (1.04, 1.53) | 1.27 (1.05, 1.54) | 1.28 (1.06, 1.55) |
| Entrepreneurs | 235 | 1.82 (1.07, 3.12) | 1.82 (1.06, 3.12) | 1.95 (1.14, 3.34) |
| Physical activity |  | p*_interaction_*= 0.012 | p*_interaction_*= 0.007 | p*_interaction_*= 0.002 |
| No or mild | 1350 | 1.17 (0.97, 1.42) | 1.09 (0.90, 1.32) | 1.16 (0.96, 1.40) |
| High | 560 | 1.28 (0.93, 1.76) | 1.67 (1.21, 2.29) | 1.66 (1.21, 2.28) |
| N. chronic diseases |  | p*_interaction_*= 0.705 | p*_interaction_*= 0.477 | p*_interaction_*= 0.886 |
| <3 | 815 | 1.31 (0.95, 1.80) | 1.11 (0.81, 1.52) | 1.33 (0.97, 1.83) |
| ≥3 | 1219 | 1.22 (1.02, 1.47) | 1.30 (1.08, 1.56) | 1.32 (1.09, 1.58) |
| MMSE |  | p*_interaction_*= 0.031 | p*_interaction_*= 0.101 | p*_interaction_*= 0.021 |
| <28 | 180 | 2.03 (1.21, 3.40) | 1.78 (1.08, 2.94) | 2.08 (1.26, 3.43) |
| ≥28 | 1852 | 1.17 (0.99, 1.39) | 1.19 (1.00, 1.41) | 1.24 (1.05, 1.47) |
| Cardiovascular diseases |  | p*_interaction_*= 0.243 | p*_interaction_*= 0.144 | p*_interaction_*= 0.202 |
| No | 1667 | 1.31 (1.09, 1.58) | 1.33 (1.10, 1.59) | 1.40 (1.17, 1.69) |
| Yes | 367 | 1.08 (0.79, 1.48) | 1.17 (0.84, 1.62) | 1.23 (0.89, 1.70) |
| Cancer |  | p*_interaction_*= 0.322 | p*_interaction_*= 0.693 | p*_interaction_*=0.868 |
| No | 1867 | 1.29 (1.09, 1. 52) | 1.25 (1.06, 1.48) | 1.35 (1.14, 1.59) |
| Yes | 167 | 1.01 (0.60, 1.72) | 1.56 (0.93, 2.61) | 1.40 (0.83, 2.38) |
| Respiratory diseases |  | p*_interaction_*= 0.500 | p*_interaction_*= 0.074 | p*_interaction_*=0.125 |
| No | 1858 | 1.24 (1.05, 1.46) | 1.20 (1.01, 1.41) | 1.28 (1.08, 1.51) |
| Yes | 176 | 1.24 (0.73, 2.13) | 1.74 (1.00, 3.01) | 1.72 (1.00, 2.98) |

*Abbreviations*: HR, hazard ratio; 95%CI, 95% Confidence Interval; NOx, nitrogen oxides; PM2.5, Particular Matter with a diameter not exceeding 2.5 μm; PM10, Particular Matter with a diameter not exceeding 10 μm. *Notes*. Model is adjusted for age, sex, educational level, occupation, smoking habits, and alcohol consumption.

**Supplementary Table 5. Difference according to exposure level in changes in standardized calf circumference, handgrip, chair stand test, and walking speed test results over 12** years (linear mixed models)

|  | **Difference in change by year (95% CI)**  ***p-value*** | | |
| --- | --- | --- | --- |
|  | **NOx**  **(> vs ≤32 μg/m^3^)** | **PM2.5**  **(> vs ≤8.29 μg/m^3^)** | **PM10**  **(> vs ≤14.7 μg/m^3^)** |
| Calf circumference (cm/year, n=2328) |  |  |  |
| *Model 1* | -0.01 (-0.03, 0.01)  p=0.207 | -0.01 (-0.01, 0.0002)  p=0.058 | -0.01 (-0.01, -0.001)  p=0.017 |
| *Model 2* | -0.004 (-0.01, 0.002)  p=0.217 | -0.02 (-0.04, 0.001)  p=0.059 | -0.01 (-0.01, -0.001)  p=0.017 |
| Handgrip (N/year, n=1848) |  |  |  |
| *Model 1* | -0.003 (-0.01, 0.001)  p=0.179 | -0.001 (-0.005, 0.004)  p=0.791 | -0.003 (-0.01, 0.001) p=0.150 |
| *Model 2* | -0.003 (-0.01, 0.001)  p=0.179 | -0.001 (-0.005, 0.004)  p=0.795 | -0.003 (-0.01, 0.001) p=0.150 |
| Chair stand test (s, n=2347) |  |  |  |
| *Model 1* | 0.01 (0.01, 0.02)  p<0.001 | 0.01 (0.01, 0.02)  p=0.001 | 0.02 (0.01, 0.02)  p<0.001 |
| *Model 2* | 0.01 (0.01, 0.02)  p<0.001 | 0.01 (0.01, 0.02)  p<0.001 | 0.02 (0.01, 0.02)  p<0.001 |
| Walking speed (m/s, n=2346) |  |  |  |
| *Model 1* | -0.01 (-0.01, -0.002)  p=0.008 | -0.01 (-0.01, -0.002)  p=0.008 | -0.01 (-0.01, -0.002)  p=0.012 |
| *Model 2* | -0.01 (-0.02, -0.003)  p=0.006 | -0.01 (-0.02, -0.003)  p=0.006 | -0.01 (-0.01, -0.002)  p=0.008 |

*Abbreviations*: 95%CI, 95% Confidence Interval; NOx, nitrogen oxides; PM2.5, Particular Matter with a diameter not exceeding 2.5 μm; PM10, Particular Matter with a diameter not exceeding 10 μm. *Notes*. Model 1 is adjusted for age and sex; Model 2 is adjusted also for educational level, occupation, smoking habits, and alcohol consumption. Muscle parameters have been standardized and transformed to z-scores. Time is included in the model in years, i.e. β coefficients represent the difference in standard deviations of the annual change of muscle parameters between participants with higher vs lower pollutant exposures.

**Supplementary references**

S1. Hu Z, Tian Y, Song X, Zeng F, Yang A. Associations between indoor air pollution for cooking and heating with muscle and sarcopenia in Chinese older population. Journal of Cachexia, Sarcopenia and Muscle. 2023;14:2029-43. doi:https://doi.org/10.1002/jcsm.13281

S2. Jiang M, Ren X, Han L, Ma T, Zheng X. Association between Household Solid Fuel Use and Sarcopenia Risk among Middle-Aged and Older Adults in China: A Nationwide Population-Based Cohort Study. J Nutr Health Aging. 2023;27:472-8. doi:10.1007/s12603-023-1933-x

S3. Su S, Zhou Y, Wang K, Liu A, Lei L, Ma H, et al. Effects of household solid fuel use on sarcopenia in middle-aged and older adults: evidence from a nationwide cohort study. Front Public Health. 2024;12:1337979. doi:10.3389/fpubh.2024.1337979

S4. Li Y, Ou X, Ou Q, Pan D. Air pollution and risk of sarcopenia: A two-sample Mendelian randomized study. Chemosphere. 2024;351:141145. doi:10.1016/j.chemosphere.2024.141145

S5. Newman AB, Kupelian V, Visser M, Simonsick EM, Goodpaster BH, Kritchevsky SB, et al. Strength, but not muscle mass, is associated with mortality in the health, aging and body composition study cohort. J Gerontol A Biol Sci Med Sci. 2006;61:72-7. doi:10.1093/gerona/61.1.72

S6. O'Bryant SE, Humphreys JD, Smith GE, Ivnik RJ, Graff-Radford NR, Petersen RC, et al. Detecting Dementia With the Mini-Mental State Examination in Highly Educated Individuals. Archives of Neurology. 2008;65:963--7 , pmid = 18625866. doi:10.1001/archneur.65.7.963

S7. Caldern-Larraaga A, Vetrano DL, Onder G, Gimeno-Feliu LA, Coscollar-Santaliestra C, Carf. Assessing and Measuring Chronic Multimorbidity in the Older Population: A Proposal for Its Operationalization. The Journals of Gerontology Series A: Biological Sciences and Medical Sciences. 2016;72:1417--23 , pmid = 28003375. doi:10.1093/gerona/glw233

S8. Wang M, Hu L, Peng H, Yao J, Zhang X, Zhang Z. The longitudinal association between indoor air pollution and sarcopenia in China: the mediating role of depression. Environ Sci Pollut Res Int. 2023;30:115506-16. doi:10.1007/s11356-023-30379-x

S9. Liu X, Li Y, Li F, Yang W, Ji W, Chen N, et al. Exposure to air pollution, genetic susceptibility, and prevalence of sarcopenia in the UK. Ecotoxicology and Environmental Safety. 2024;285:117143. doi:https://doi.org/10.1016/j.ecoenv.2024.117143

S10. Guralnik J, Bandeen-Roche K, Bhasin SAR, Eremenco S, Landi F, Muscedere J, et al. Clinically Meaningful Change for Physical Performance: Perspectives of the ICFSR Task Force. J Frailty Aging. 2020;9:9-13. doi:10.14283/jfa.2019.33
